# Functional beliefs and risk minimizing beliefs among Thai healthcare workers in Maharaj Nakorn Chiang Mai hospital: its association with intention to quit tobacco and alcohol

**DOI:** 10.1186/s13011-017-0118-1

**Published:** 2017-07-12

**Authors:** Surin Jiraniramai, Wichuda Jiraporncharoen, Kanokporn Pinyopornpanish, Nalinee Jakkaew, Tinakon Wongpakaran, Chaisiri Angkurawaranon

**Affiliations:** 1 Department of Family Medicine, Faculty of Medicine, Chiang Mai University, 110 Intawaroros Road, Sriphum, Muang, Chiang Mai, 50200 Thailand; 2 Department of Psychiatry, Faculty of Medicine, Chiang Mai University, Chiang Mai, Thailand

## Abstract

**Background:**

Individual health beliefs are likely to play a key role in how people respond to knowledge and information about the potential harm from smoking and alcohol abuse. The objectives of the study were to 1) explore whether functional beliefs and risk minimizing beliefs were associated with intention to quit smoking and confidence to quit smoking and 2) explore whether functional beliefs and risk minimizing beliefs were associated with intention to quit alcohol drinking and confidence to quit alcohol drinking.

**Methods:**

A cross-sectional survey was conducted in 2013 among health care workers working in Thailand. Using predicted factor scores from factor analysis, the relationship between factor scores for each of the two beliefs and intention to quit and confidence to quit were tested using ANOVA and further adjusted for age and sex using linear regression.

**Results:**

Functional beliefs were inversely associated with the intention to quit and confidence to quit smoking. Both functional beliefs and risk minimizing beliefs were each inversely associated with the intention to quit and confidence to quit alcohol drinking.

**Conclusion:**

Our study enhances the understanding of the complexities of health beliefs regarding these two commonly abused substances. As functional beliefs were associated with smoking and alcohol use, interventions to counter the cultural values and individual beliefs about the benefits of smoking and alcohol use are needed. Tackling risk minimizing beliefs by providing individualized feedback regarding harm may also be useful in alcohol drinkers.

**Electronic supplementary material:**

The online version of this article (doi:10.1186/s13011-017-0118-1) contains supplementary material, which is available to authorized users.

## Background

The prevalence of alcohol drinking and smoking is high globally and evidence has shown that these risky health behaviors may lead to many negative consequences, with the risk increasing when taking them together [1–3]. The Thai national health surveys have reported that both the prevalence of alcohol drinking and smoking have been steady from 2004 but had minimally increased by 2011 [4, 5]. Literature suggests that each year, a small proportion of people have reported trying to stop smoking (7%) or drinking (9%) [4, 6, 7]. Knowledge of the negative effects was mentioned as one of the main reason why people stop smoking. However, many already know that excessive alcohol consumption and smoking can be addictive and can cause serious health and social harm [8]. This suggests that people respond to this knowledge differently and often people continue to smoke and drink despite knowing the potential harm.

Individual health beliefs are likely to play a key role in how people respond to such knowledge and information. People try to make a positive change when they perceive that their current behavior may lead to a negative outcome. However, the barrier against this positive force is the unsatisfying feeling when trying to avoid their previous negative behavior or when they are unable to resist and revert to their old negative behavior. The psychological tension created when the individual’s negative behavior conflicts with their belief is known as cognitive dissonance [9]. If over time they could not resist their own desire and are unable to make positive changes, often it is their mindset or beliefs that are changed instead [10].

Giving up addictive behaviors, such as smoking, is often difficult. Many individuals may change their attitude, which is the path of least resistance, by adopting other beliefs to help reduce cognitive dissonance [11]. Health beliefs commonly found among alcohol drinkers and smokers that help minimize cognitive dissonance can be divided into two types, risk minimizing beliefs and functional beliefs. People use risk minimizing beliefs to help alleviate the seriousness of the problem by perceiving that, for them, there is less opportunity to experience any negative effects from that behavior or by minimizing the negative feature of the undesirable consequences of that behavior. An example of risk minimizing belief is the idea that ‘the harms or problems associated with smoking and drinking does not apply to me’ [12, 13]. Functional beliefs are related to the perceived benefits of the behavior or beliefs in the value of the behavior. For example, many smokers may feel that smoking ‘is effective for reducing stress and increase concentration’ [14, 15].

Functional beliefs and risk minimizing beliefs about smoking and smoking motives have been examined since the late 1960s where researchers have developed scales for assessing these beliefs [16]. Later studies examined the associations between these two types of beliefs and smoking. Studies, including one from Thailand, have shown a strong association between risk-minimizing and current smoking [17, 18]. Smokers often normalized and minimized the dangers of smoking. In addition, evidence from Thailand and other countries, have found that risk minimizing beliefs were also associated with a reduced intention to quit smoking [12, 19, 20] and confidence to quit smoking [21, 22] . The same direction of association was found for functional beliefs [18, 23]. The association, however, between health beliefs and smoking may vary due to differences in sociocultural factors and norms in each setting [21]. Moreover, unlike smoking, only few research studies have explored the association between risk minimizing beliefs and the function of beliefs and alcohol use [7, 15, 24] despite the fact of the correlation between both behaviors [25] .

Our study aimed to 1) explore whether functional beliefs and risk minimizing beliefs were associated with the intention to quit smoking and confidence to quit smoking in Thailand and 2) explore whether functional beliefs and risk minimizing beliefs were associated with the intention to quit alcohol drinking and confidence to quit alcohol drinking in Thailand. Exploring these two beliefs with both smoking and alcohol use may increase the understanding of the complexities of health beliefs regarding these two commonly abused substances.

## Methods

A cross-sectional survey was conducted among health care workers in a University Hospital in Chiang Mai, Thailand in 2013. A detailed description of the survey has been published [26]. In summary, 3204 participants (59.7% response rate) completed a self-administered online questionnaire on smoking and alcohol use as well as their beliefs about smoking and alcohol use.

### Health beliefs

Health beliefs about smoking and alcohol drinking were evaluated separately using a nine-item questionnaire derived from previous literature [18, 19]. The first five items were related to functional beliefs of smoking or alcohol use. The last four items referred to risk minimizing beliefs about the risk and harms of smoking or alcohol use (Tables 1, 2). Participants rated their agreement with each functional and risk minimizing belief. Agreement scores ranged from one to five. A score of five indicated that the participant totally agreed with the statement and a score of one indicated that the participant totally disagreed with the statement.

**Table 1:** Health beliefs and intention to quit smoking among current smokers and recent quitters

Beliefs	Loading Factor	% Agree	Mean Level of Agreement					df, test value	*p*-value
			No Intention to Quit (77)	Intend to Quit in >6 Months (49)	Intend to Quit in <6 Months (15)	Intend to Quit in 1 Month (26)	Recent Quitters (20)		
Factor 1 Functional (α = 0.95)									
You enjoy smoking too much to give it up.	0.88	12.3	2.6	2.3	2.9	1.8	1.5	4, 22.9	<0.01
Smoking calms you down when you are stressed or upset.	0.77	33.7	3.1	3.0	3.5	2.5	2.0	4, 18.1	0.01
Smoking helps you concentrate better.	0.91	13.9	2.6	2.3	2.9	1.7	1.8	4, 18.2	<0.01
Smoking is an important part of your life.	0.87	9.6	2.5	2.0	2.6	1.3	1.5	4, 30.6	<0.01
Smoking makes it easier for you to socialize.	0.78	8.0	2.3	1.7	2.3	1.3	1.6	4,18.5	<0.01
Factor 2 Risk Minimizing (α = 0.88)									
The medical evidence that smoking is harmful is exaggerated.	0.82	43.8	3.3	3.1	3.1	3.4	2.3	4, 8.81	0.06
Smoking is no riskier than lots of other things that people do.	0.81	19.8	2.8	2.5	2.6	2.0	2.0	4, 11.1	0.03
You must die of something, so why not enjoy yourself and smoke.	0.56	15.0	2.6	2.3	2.5	1.9	1.9	4, 8.96	0.06
I think I must have the sort of good genes that means I can smoke without getting any harm.	0.57	9.6	2.4	2.1	2.9	1.7	1.6	4, 17.7	<0.01

Agreement scores ranged from 1 to 5. A score of 5 indicated that the participant totally agreed with the statement, a score of 4 indicated that the participant somewhat agreed with the statement, a score of 3 reflected that the participant was unsure about the statement, a score of 2 and a score of 1 indicated that the participant somewhat disagree and totally disagree with the statement respectively. df = degree of freedom, Test statistic and *p*-value obtained from Kruskal-Wallis equality-of-population rank test

**Table 2:** Health beliefs and intention to quit alcohol drinking among current drinkers and recent quitters

Beliefs	Loading Factor	% Agree	Mean Level of Agreement					df, test value	*p*-value
			No Intention to Quit (77)	Intend to Quit in >6 Months (49)	Intend to Quit in <6 Months (15)	Intend to Quit in 1 Month (26)	Recent Quitters (20)		
Factor 1 Functional (α = 0.69)									
You enjoy drinking too much to give it up.	0.43	8.1	2.2	2.2	2.2	1.9	1.3	4, 313.6	<0.01
Drinking calms you down when you are stressed or upset.	0.55	19.5	2.8	2.8	2.9	2.3	1.4	4, 444.3	<0.01
Drinking helps you concentrate better.	0.50	3.8	1.8	1.8	1.8	1.5	1.2	4, 156.0	<0.01
Drinking is an important part of your life.	0.50	2.9	1.7	1.7	.16	1.4	1.2	4, 143.3	<0.01
Drinking makes it easier for you to socialize.	0.35	19.5	2.8	2.8	2.7	2.4	1.5	4, 368.	<0.01
Factor 2 Risk Minimizing (α = 0.60)									
The medical evidence that drinking is harmful is exaggerated.	0.68	29.0	2.8	2.7	2.6	2.5	2.2	4, 64.5	<0.01
Drinking is no riskier than lots of other things that people do.	0.78	13.1	2.4	2.3	2.3	2.1	1.8	4, 92.9	<0.01
You must die of something, so why not enjoy yourself and drink.	0.80	11.1	2.4	2.3	2.3	2.0	1.4	4, 274.1	<0.01
I think I must have the sort of good genes that means I can drink without any harm.	0.63	5.9	2.2	2.1	2.1	1.9	1.4	4, 182.2	<0.01

Agreement scores ranged from 1 to 5. A score of 5 indicated that the participant totally agreed with the statement, a score of 4 indicated that the participant somewhat agreed with the statement, a score of 3 reflected that the participant was unsure about the statement, a score of 2 and a score of 1 indicated that the participant somewhat disagree and totally disagree with the statement respectively. df = degree of freedom, Test statistic and *p*-value obtained from Kruskal-Wallis equality-of-population rank test

### Measures of intention to quit

The history of each substance used was initially categorized into four groups. The first group was for those who had never used substances. The second group was those who were former users defined as having stopped using the substance for longer than one year. The third group was those who were recent quitters defined as having recently stopped within one year and the last group of users were those who categorized themselves as currently using the substance (within the past 3 months). If participants were currently using a particular substance, they were asked about their intention to quit each particular substance using a four-item response: 1-no intention to quit, 2-intention to quit in the next six months, 3-intention to quit within six months and 4-intent to quit within 30 days. If participants indicated that they planned on give up a particular substance, they were also asked about their confidence to quit each particular substance by using a five-item response of success: 0-not at all confident, 1-not confident (< 25% chance of success), 2-moderately confident (25-50% chance of success), 3-confident (50-75% chance of success and 4-very confident (> 75% chance of success).

### Data analysis

Participants who were current users and those who had stopped within the past year were used in the analysis for each substance. Separately for tobacco and alcohol consumption, a factor analysis with orthogonal rotation (varimax) was used to examine whether the nine-item questionnaires formed the two coherent health beliefs (functional beliefs and risk minimizing beliefs) as hypothesized. A loading factor of at 0.4 was used as a cutoff point [27]. Kruskal-Wallis test was used to determine the association between the agreement score for each item with intention to quit. Using predicted factor scores from factor analysis, the relationship between factor scores for each of the two beliefs and intention to quit and confidence to quit were tested using ANOVA. These associations were further adjusted for age and sex using linear regression, as it was an a-priori belief that these two factors could be considered as potential confounders. Lastly, functional belief scores and risk minimizing belief scores were categorized into quartiles and its association with recent cessation of smoking and alcohol use using logistic regression were examined. Sensitivity analyses were performed by excluding recent quitters from the analyses. A *p*-value of ≤0.05 was considered statistically significant. All analyses were conducted using STATA version 12.0.

## Results

Of the 3204 participants, 20 participants had recently quit smoking within a year and 167 were current smokers (5.2%). It was these 187 smokers and recent quitters that are used for further analysis on health beliefs about smoking and intention to quit smoking (Table 1). The vast majority of the 187 smokers and recent quitters were male, only seven female were current smokers (Additional file 1: Table S1). For alcohol use, 572 participants stated that they had recently stopped drinking alcohol within one year while 992 were current alcohol drinkers. It is these 1564 participants that are used for further analysis on health beliefs about drinking and intention to quit alcohol drinking (Table 2). Of the 1564 participants who were current drinkers or had recently given up alcohol, 995 were females and 78 were males (Additional file 1: Table S2).

### Health beliefs and intention to quit smoking and confidence to quit smoking

Using factor analysis with orthogonal rotation (varimax), there were four potential factors that could be derived from the nine-item questionnaire. However, only in the first two factors the individual items had a factor loading of greater than 0.4 (Additional file 1: Table S3), thus supporting that the nine-items form two types of coherent beliefs (Table 1). Results suggest that current smokers and recent quitters engaged in a number of functional and risk minimizing beliefs about smoking. The most common functional belief was that “Smoking calms you down when you are stressed or upset”, found in about 33.7% of smokers and recent quitters. The most common risk minimizing belief was that “The medical evidence that smoking is harmful is exaggerated”, was found in 43.8% of smokers and recent quitters (Additional file 1: Table S4). Results from Table 1 suggest that each of the functional beliefs were associated with intention to quit. Higher agreement in each functional belief about smoking was inversely associated with the intention to quit. Only three of the four risk-minimizing beliefs were associated with intention to quit (Table 1).

**Fig. 1:**
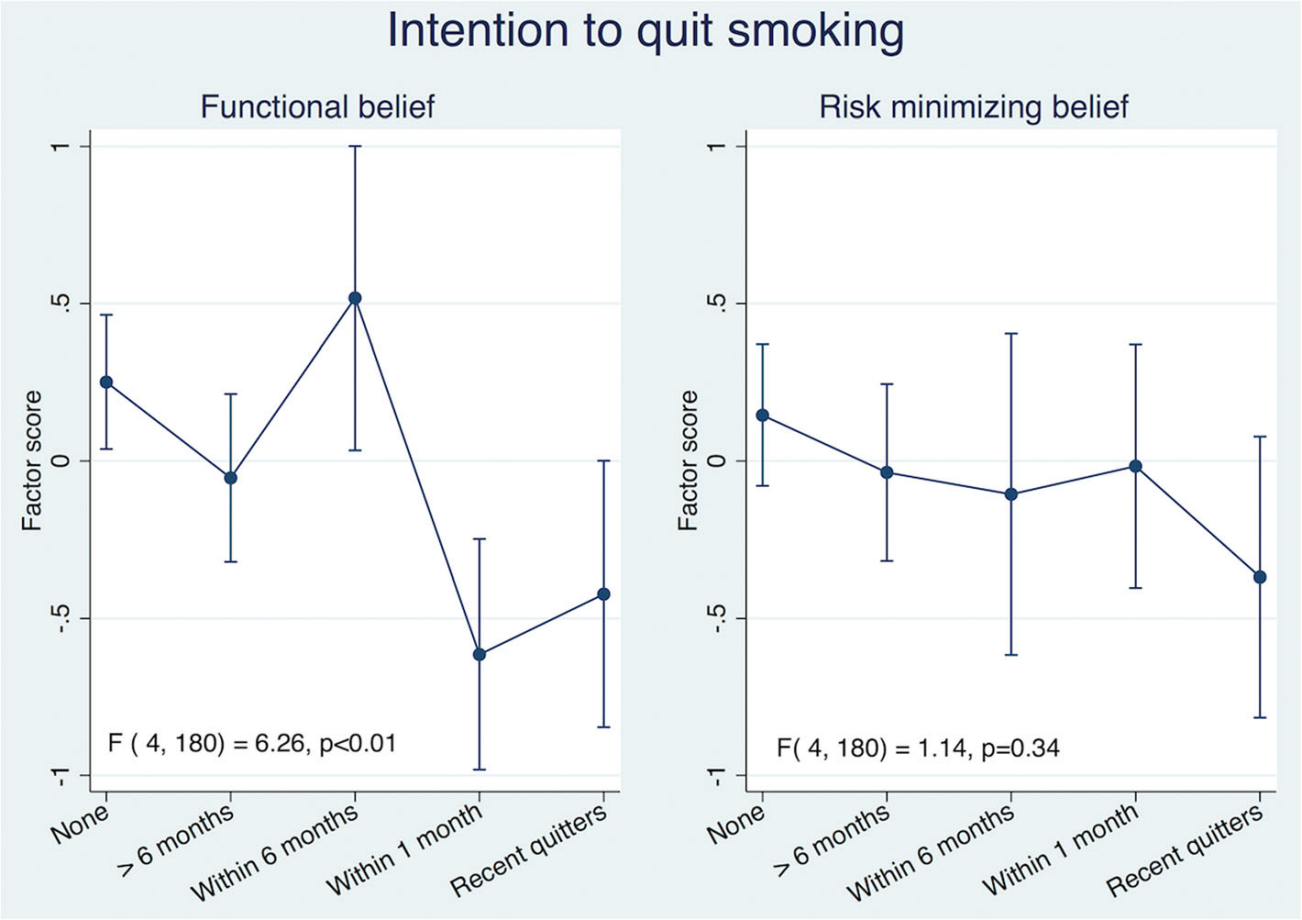
Health Beliefs and Intention to Quit Smoking. Results are adjusted for age and sex. Higher factor score indicate higher level/agreement of belief. Vertical lines represents 95% confidence intervals. *P*-values obtained from values of the F statistic and the corresponding degrees of freedom

**Fig. 2:**
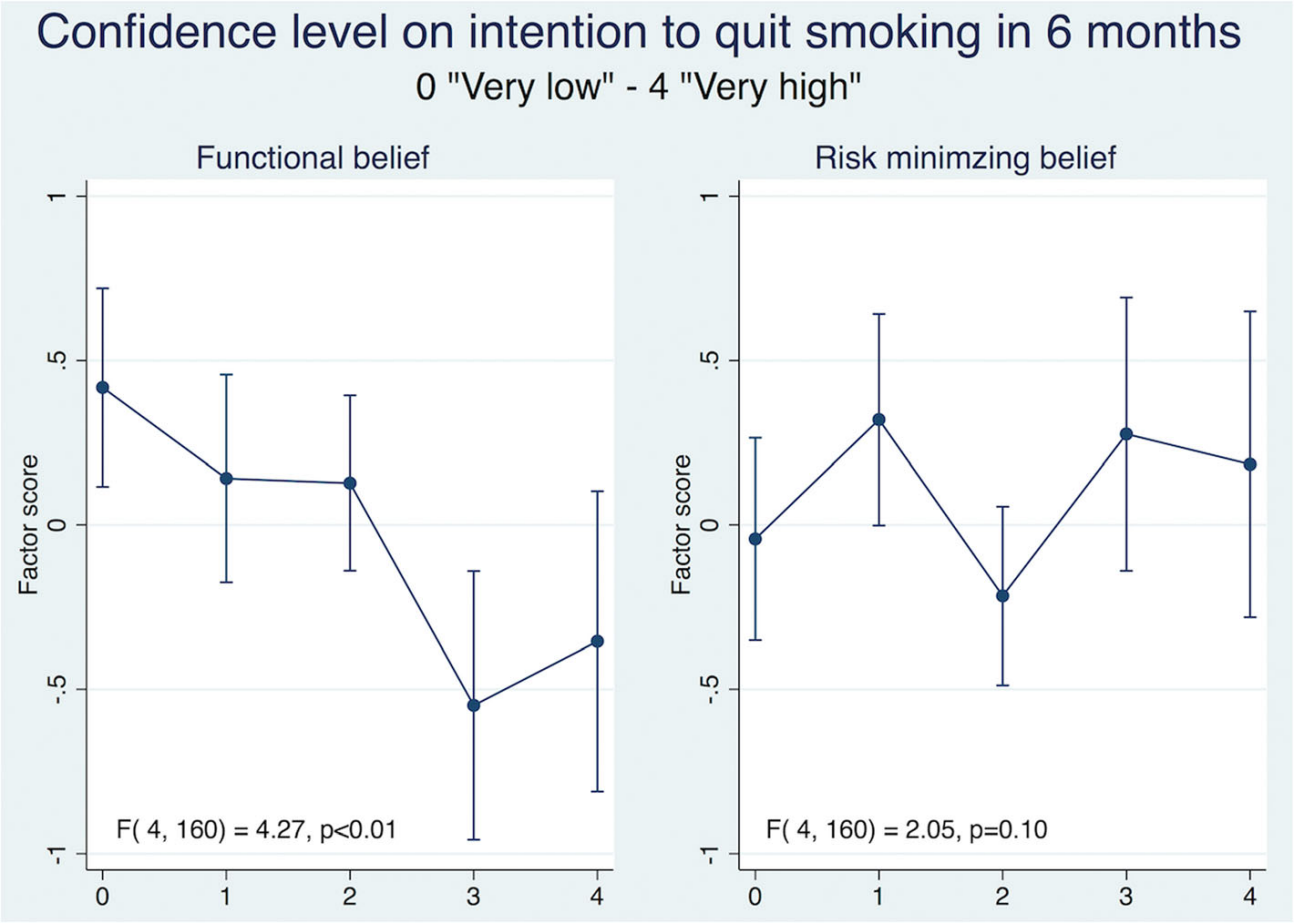
Health Beliefs and Confidence Level on the Intention to Quit Smoking. Results are adjusted for age and sex. Confidence to quit smoking was assessed by using a five-item response of success: 0-not at all confident, 1-not confident (< 25% chance of success), 2-moderately confidently (25-50% chance of success), 3-confident (50-75% chance of success and 4-very confident (> 75% chance of success). Vertical lines represents 95% confidence intervals. *P*-values obtained from values of the F statistic and the corresponding degrees of freedom

Using factor scores derived from factor analysis, only the factor score for functional beliefs was associated with intention to quit smoking and the confidence level in quitting. Adjusted for age and sex, individuals with higher functional beliefs of smoking were less likely to quit smoking (Fig. 1) and were less confident of being able to quit (Fig. 2). The factor score for risk minimizing beliefs was not associated with intention to quit smoking (Fig. 1) and the confidence level in quitting (Fig. 2). The associations with intention to quit did not materially change when recent quitters were excluded from the analyses (Additional file 2: Figure S1).

In a multivariate regression model, there was some weak evidence of a gradient in associations between quartiles of functional belief scores and recent smoking cessation (Wald test statistic = 3.02,df = 1, *p* = 0.08) as well as between quartiles of risk minimizing belief scores and recent smoking cessation (Wald test statistic = 6.35,df = 1, *p* = 0.02). Those with functional beliefs scores and risk minimizing scores in the highest quartile were less likely to have recently quit smoking (Table 3).

**Table 3:** Association between functional belief and cessation of smoking within past year

	Adjusted Odds ratio	95% CI	test value	df	*p*-value
Functional belief factor score (quartile)			3.02	1	0.08
1st (lowest)	Reference				
2nd	1.97	0.34 to 11.2			
3rd	0.58	0.08 to 4.03			
4th (highest)	0.42	0.05 to 3.57			
Risk minimizing belief factor score (quartile)			6.35	1	0.02
1st (lowest)	Reference				
2nd	0.55	0.15 to 2.04			
3rd	---	---			
4th (highest)	0.38	0.08 to 1.75			
Age (increase)	1.01	0.95 to 1.07	0.14	1	0.71
Sex			3.60	1	0.06
Female	Reference				
Male	0.17	0.03 to 1.06			
Income (baht/month)			4.32	2	0.11
< 30,000	Reference				
30,000-60,000	3.87	0.67 to 22.3			
> 60,000	5.24	0.77 to 35.6			
Highest education			4.52	1	0.03
Below Bachelor’s degree	Reference				
Bachelor’s degree	0.17	0.03 to 0.87			
Higher than Bachelor’s degree	---	---			
Occupation			0.29	1	0.59
Health professional	Reference				
Non-health professional	0.67	0.17 to 2.72			

Test value using Walds test; *CI* Confidence interval; *df* degree of freedom; all odds ratios are adjusted for all variables presented in the table; empty cells indicate that there are no observations

### Health beliefs and intention to quit drinking and confidence to quit drinking

**Fig. 3:**
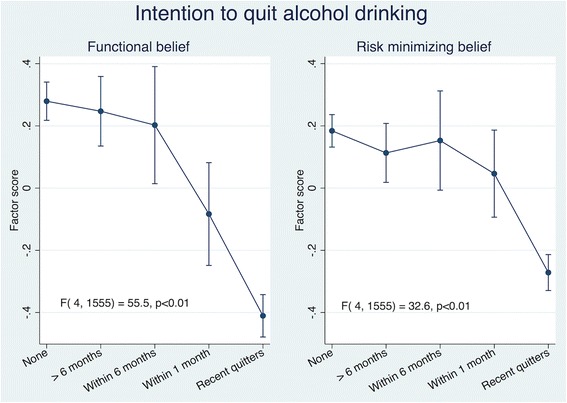
Health Beliefs and Intention to Quit Alcohol Drinking. Results are adjusted for age and sex. Higher factor score indicate higher level/agreement of belief. Vertical lines represents 95% confidence intervals. *P*-values obtained from values of the F statistic and the corresponding degrees of freedom

**Fig. 4:**
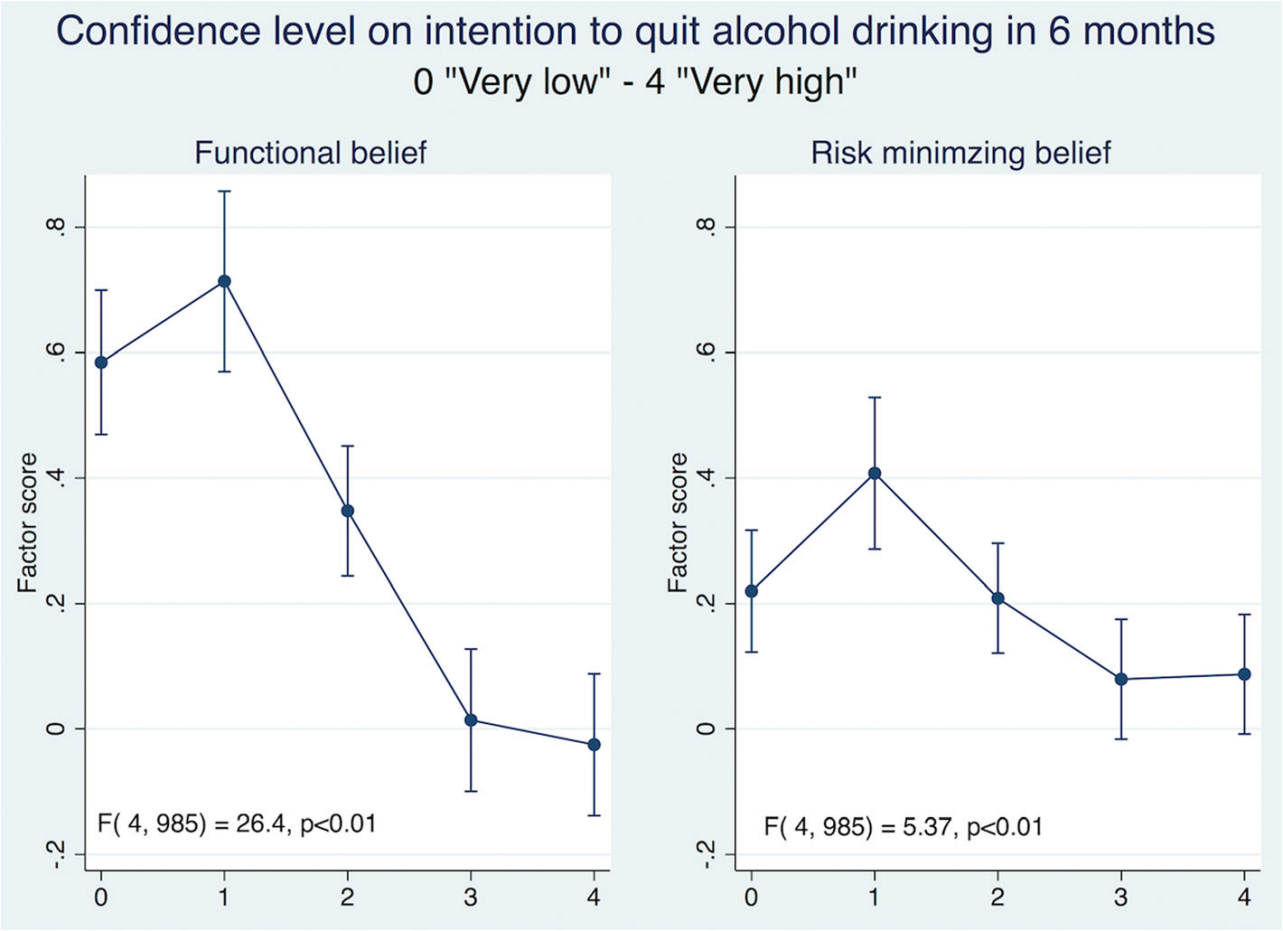
Health Beliefs and Confidence Level on Intention to Quit Alcohol Drinking. Results are adjusted for age and sex. Confidence to quit alcohol drinking was assessed by using a 5-item response of success: 0-not at all confident, 1-not confident (< 25% chance of success), 2-moderately confidently (25-50% chance of success), 3-confident (50-75% chance of success and 4-very confident (> 75% chance of success). Vertical lines represents 95% confidence intervals. *P*-values obtained from values of the F statistic and the corresponding degrees of freedom

Similar to results for smoking, although there were four potential factors that could be derived from the questionnaire (Additional file 1: Table S5), the loading factors were aggregated towards two types of coherent beliefs (Table 2). For functional beliefs, approximately 20% agreed that “Drinking calms you down when you are stressed or upset” and that “Drinking makes it easier for you to socialize”. The most common risk minimizing belief was that Medical evidence that drinking is harmful is exaggerated” (Additional file 1: Table S6). Displaying a similar pattern to the intention to quit smoking, higher agreement in each of the functional beliefs of alcohol was inversely associated with the intention to quit (Table 2). All four risk minimizing beliefs of alcohol were associated with an intention to quit alcohol (Table 2). Adjusting for age and sex, factor scores of functional beliefs and risk minimizing beliefs were each inversely associated with intention to quit alcohol drinking (Fig. 3) and confidence to quit drinking (Fig. 4). Those with higher scores in functional and risk minimizing beliefs about drinking were less likely to quit drinking and had a lower confidence in quitting. Some power was loss in the sensitivity analysis but the associations with intention to quit did not materially change when recent quitters were excluded from the analyses (Additional file 3: Figure S2).

In a multivariate regression model, there was a gradient in associations between quartiles of functional belief scores and recent cessation of alcohol use as well as between quartiles of risk minimizing belief scores and recent cessation of alcohol use. Compare to those in lowest quartile of functional belief scores, the odds ratio for recent cessation of alcohol use was 0.22 (95% CI 0.15 to 0.33) for those with score in the 2nd quartile, 0.11 (95% CI 0.07 to 0.16) for those in the 3rd quartile and 0.04 (95% CI 0.03 to 0.07) for those with the highest functional belief scores (Wald test statistic = 178.2,df = 1, *p* < 0.01). This gradient was also demonstrated with risk minimizing beliefs scores. Compared to those in the lowest quartile of risk minimizing beliefs scores, the odds ratio for recent cessation of alcohol use was 0.50 (95% CI 0.34 to 0.74) for those with score in the 2nd quartile, 0.32 (95% CI 0.21 to 0.48) for those in the 3rd quartile and 0.21 (95% CI 0.13 to 0.32) for those with the highest functional belief scores (Wald test statistic = 53.4,df = 1, *p* < 0.01) (Table 4).

**Table 4:** Association between functional belief, risk minimizing beliefs and cessation of alcohol use within past year

	Adjusted Odds ratio	95% CI	test value	df	*p*-value
Functional belief factor score (quartile)			178..2	1	<0.01
1st (lowest)	Reference				
2nd	0.22	0.15 to 0.33			
3rd	0.11	0.07 to 0.16			
4th (highest)	0.04	0.03 to 0.07			
Risk minimizing belief factor score (quartile)			53.4	1	<0.01
1st (lowest)	Reference				
2nd	0.50	0.34 to 0.74			
3rd	0.32	0.21 to 0.48			
4th (highest)	0.21	0.13 to 0.32			
Age (increase)	1.03	1.01 to 1.04	13.2	1	<0.01
Sex			65.6	1	<0.01
Female	Reference				
Male	0.26	0.18 to 0.36			
Income (baht/month)			4.93	2	0.08
< 30,000	Reference				
30,000-60,000	1.34	0.96 to 1.86			
> 60,000	1.54	1.01 to 2.36			
Highest education			7.11	2	0.03
Below Bachelor’s degree	Reference				
Bachelor’s degree	1.43	1.01 to 2.02			
Higher than Bachelor’s degree	1.93	1.16 to 3.22			
Occupation			3.98	1	0.05
Health professional	Reference				
Non-health professional	0.72	0.53 to 0.99			

Test value using Walds test; *CI* confidence interval; *df* degree of freedom; all odds ratios are adjusted for all variables presented in the table

## Discussion

Using factor analysis, our nine-item questionnaires form two coherent beliefs about smoking and alcohol use, the “functional belief” and the “risk-minimizing belief”. Results suggested that, the functional beliefs were associated with the intention to quit and confidence to quit smoking. While both functional beliefs and risk minimizing beliefs were associated with the intention to quit and confidence to quit alcohol drinking.

### Function beliefs, risk minimizing beliefs and smoking

When considering the intention and confidence to quit smoking*,* the highest agreement on risk minimizing beliefs from our sample was the skeptical belief that “Medical evidence that smoking is harmful is exaggerated”. This result was similar to a previous study in Australia [19] which reflected lack of understanding in the harm or health outcomes from smoking and its consequences. This belief can usually be found in the smokers, which was also reflected in our study. However, using factor scores, our study did not find an association between risk minimizing beliefs and intention to quit and confidence to quit smoking. Risk minimizing beliefs can be considered “weak beliefs” as they can be easily influenced and changed [28]. This potential fluctuation in risk minimizing beliefs and our small sample of current smokers and recent quitters may be reasons why this study could not detect any association between risk minimizing beliefs and the intention to stop smoking.

Higher agreement in functional beliefs about smoking was inversely associated with intention to quit and confidence to quit smoking, which has been observed in a previous study [29]. Smokers have dissonant reduction when they attempt to enhance the functional beliefs in smoking [18]. When exploring each functional belief individually, the functional belief that was most commonly found was that “Smoking can calm you down when you are stressed or upset”. This is not surprising as nicotine can modulate pathways involved in stress response, depression and anxiety [30]. Our study found that the few smokers had functional beliefs about ‘Smoking for social enhancement’. This is potentially due to the regulatory environment in Thailand that restricts smoking areas and has banned smoking advertisements on television and radio [20].

### Functional beliefs, risk minimizing beliefs and alcohol use

Similar to results for smoking, this study found an inverse association between functional beliefs and the intention to quit alcohol drinking. This may be because concurrent use of alcohol and smoking are common [25]. However, when exploring each functional belief individually, there were different patterns of functional beliefs between smoking and alcohol drinking. Firstly, in contrast to smoking, functional beliefs of drinking for “social enhancement” was quite common. In Thailand, alcohol use is integrated into social norms and also traditional rites. In additional, drinking is rarely perceived as a social problem [31, 32].

Risk minimizing beliefs about drinking were significantly associated with the intention to quit and confidence to quit alcohol drinking, while this association was not found in smoking. Currently drinkers tend to have risk minimizing beliefs as most of Thai people who use alcohol are at low to moderate risk of harm, which may not show the serious health outcomes [33, 34]. Furthermore, there is conflicting evidence regarding the potential protective effect of alcohol against coronary heart disease [34, 35], which may be why risk minimizing beliefs are associated with alcohol use and inversely associated with the intention to quit.

The present study has some limitations. The response rate of our study was 60%, which may introduce some selection bias. However, in a previous publication, we have demonstrated that our sample was representative of the source population in terms of age, sex and education level [26]. Because of the data was based on self-report, health beliefs and the intention to quit smoking and alcohol drinking may be vulnerable to social desirability bias. However, voluntary participation, assurances of confidentiality in this study may have reduced some of the impact of social desirability bias. This study was a cross-sectional data, we can only assume the temporal relationships between decreasing functional beliefs and risk minimizing beliefs with subsequent changes in willingness to quit smoking or alcohol use. However, some evidence from prospective studies of smoking have supported this notion [13, 18]. The number of smokers enrolled in this study was small, thus the estimates were imprecise and could be underpowered to detect the association between minimizing beliefs and the intention to quit and confidence to quit smoking. As other health beliefs, in particular positive or protective beliefs, were not explored in this study, we could not provide evidence on what beliefs may be protective or promotes intention to quit. Nonetheless, this study also has some strengths. Factor analysis was utilized to derive factor scores of beliefs rather than just a single question which had previously been common in past literature [36]. It is also one of the first studies to report findings for both alcohol and tobacco use.

## Conclusions

The finding that risk minimizing beliefs were associated with alcohol use and that functional beliefs were associated with both smoking and alcohol use has several implications. Risk minimizing beliefs can be overridden by giving persuasive information on the negative consequences [8]. For alcohol drinking, tools such as the Alcohol, Smoking, Substance Involvement Screening test (ASSIST) [37] which can detect and quantify the risk of harm from alcohol use may be a useful tool that can help provide individualized information and feedback.

As functional beliefs were also common, interventions to counter the cultural values and individual beliefs about the benefits of smoking and alcohol use are needed. Functional belief for stress management were commonly found. Thus smokers and alcohol drinkers ought to have or should be advised on alternative coping strategies for stress management [29]. Combined pharmacological therapy and behavioral intervention can also assist the users who have the effects of a physical addiction [38]. For functional beliefs of social enhancement attached with alcohol use, strategies employed to decrease the social value of smoking can also be applied. These may involve using classroom-based interventions, community-based strategies, and alcohol control regulations [39].

The challenges related to prevention and treatment of alcohol addiction in Thailand has been documented. This includes “poor motivation of patients” and the “belief that patients can handle problems” [40] which coincides with functional and risk minimizing beliefs explored in this study. A study from Thailand has suggested that higher level of moral beliefs and engaging in religious activities may be a protective factor of alcohol use among adolescents [41]. This is also in line with other evidence from Thailand where a few small-scale community-based approach projects have demonstrated success in addressing such issues in tackling health beliefs and substance use. Most included tailoring policies and interventions to coincide with Buddhist values and moral beliefs of the culture in the community [42–44]. This may provide a useful example for future programs.

## Additional files

Additional file 1: **Table S1.** Characteristics of current smoker and recent quitters by intention to quit status. **Table S2.** Characteristics of current drinking and recent quitters by intention to quit status. **Table S3.** Rotated factor loading on health beliefs about smoking among those with a lifetime history of smoking. **Table S4.** Distribution of respondents to each of the functional beliefs and risk minimizing beliefs of smoking. **Table S5.** Rotated factor loading on health beliefs about alcohol among those with a lifetime history of alcohol drinking. **Table S6.** Distribution of respondents to each of the functional beliefs and risk minimizing beliefs of alcohol drinking. (DOCX 99 kb)

Additional file 2: Figure S1. Sensitivity Analysis of Health Beliefs and Intention to Quit Smoking (excluding recent quitters). Results are adjusted for age and sex. Higher factor score indicate higher level/agreement of belief. Vertical lines represents 95% confidence intervals. *P*-values obtained from values of the F statistic and the corresponding degrees of freedom. (TIFF 4656 kb)

Additional file 3: Figure S2. Sensitivity Analysis of Health Beliefs and Intention to Quit Alcohol Drinking (excluding recent quitters). Results are adjusted for age and sex. Higher factor score indicate higher level/agreement of belief. Vertical lines represents 95% confidence intervals. *P*-values obtained from values of the F statistic and the corresponding degrees of freedom. (TIFF 4656 kb)
