# Supplementary material for: Functional beliefs and risk minimizing beliefs among Thai healthcare workers in Maharaj Nakorn Chiang Mai hospital: its association with intention to quit tobacco and alcohol
Source: Subst Abuse Treat Prev Policy. 2017 Jul 12;12:34. doi: 10.1186/s13011-017-0118-1 (PMC5508689; doi:10.1186/s13011-017-0118-1)
Supplement: Supplementary file 1 — Table S1. Characteristics of current smoker and recent quitters by intention to quit status. Table S2. Characteristics of current drinking and recent quitters by intention to quit status. Table S3. Rotated factor loading on health beliefs about smoking among those with a lifetime history of smoking. Table S4. Distribution of respondents to each of the functional beliefs and risk minimizing beliefs of smoking. Table S5. Rotated factor loading on health beliefs about alcohol among those with a lifetime history of alcohol drinking. Table S6. Distribution of respondents to each of the functional beliefs and risk minimizing beliefs of alcohol drinking. (DOCX 99 kb) [file 13011_2017_118_MOESM1_ESM.docx]

**Appendix Table 1 Characteristics of current smoker and recent quitters by intention to quit status**

|  | No intention to quit (n=77) | Intention to quit but in more than 6 months (n=49) | Intention to quit within 6 months (N=15) | Intention to quit within 1 month (n=26) | Recent quitters (within 1 year) (n=20) | degree of freedom | p-value |
| --- | --- | --- | --- | --- | --- | --- | --- |
| Mean Age (sd) | 39.8 (8.7) | 37.4 (9.8) | 34.7 (8.6) | 36.3 (7.9) | 41.7 (10.9) | 186 | 0.07* |
| Percent female (col %) | 3.9 | 2.0 | 6.7 | 7.7 | 15.0 | 4 | 0.25** |
| Highest education (col %) |  |  |  |  |  | 8 | 0.06** |
| Below Bachelor’s degree | 84.4 | 59.2 | 66.7 | 76.9 | 85 |  |  |
| Bachelor’s degree | 14.3 | 34.7 | 33.3 | 23.1 | 15 |  |  |
| Higher than Bachelor’s degree | 1.3 | 6.1 | 0.0 | 0 | 0 |  |  |
| Monthly income (col %) |  |  |  |  |  | 8 | 0.27** |
| <30,000 baht/month | 92.2 | 79.6 | 93.3 | 88.5 | 70 |  |  |
| 30,000-60.000 baht/month | 3.9 | 10.2 | 6.7 | 7.7 | 15 |  |  |
| >60,000 bath/month | 3.9 | 10.2 | 0 | 3.8 | 15 |  |  |
| Occupation (col %) |  |  |  |  |  | 4 | 0.35** |
| Health professionals | 15.6 | 18.4 | 13.3 | 3.8 | 25 |  |  |
| Non-health professionals | 84.4 | 81.6 | 86.7 | 96.2 | 75 |  |  |

*p-value for analysis from analysis of variance (ANOVA), ** p-value from chi-square

**Appendix Table 2 Characteristics of current drinking and recent quitters by intention to quit status**

|  | No intention to quit (n=645) | Intention to quit but in more than 6 months (n=193) | Intention to quit within 6 months (N=67) | Intention to quit within 1 month (n=87) | Recent quitters (within 1 year) (n=572) | degree of freedom | p-value |
| --- | --- | --- | --- | --- | --- | --- | --- |
| Mean Age (sd) | 38.6 (10.1) | 37.9 (10.0) | 38.1 (9.6) | 35.4 (10.0) | 41.7 (10.3) | 1563 | <0.01* |
| Percent female | 50.8 | 45.6 | 44.8 | 63.3 | 86.4 | 4 | <0.01** |
| Highest education (col %) |  |  |  |  |  | 8 | <0.01** |
| Below Bachelor’s degree | 44.0 | 47.7 | 64.2 | 40.2 | 27.5 |  |  |
| Bachelor’s degree | 48.4 | 49.7 | 34.3 | 52.9 | 55.8 |  |  |
| Higher than Bachelor’s degree | 7.6 | 2.6 | 1.5 | 6.9 | 16.7 |  |  |
| Monthly income (col %) |  |  |  |  |  | 8 | <0.01** |
| <30,000 baht/month | 66.7 | 74.1 | 79.1 | 64.4 | 44.2 |  |  |
| 30,000-60.000 baht/month | 20.3 | 20.2 | 17.9 | 25.3 | 31.9 |  |  |
| >60,000 bath/month | 13.0 | 5.7 | 3.0 | 10.3 | 23.9 |  |  |
| Occupation (col %) |  |  |  |  |  | 4 | <0.01** |
| Health professionals | 49.1 | 38.9 | 28.4 | 47.1 | 71.4 |  |  |
| Non-health professionals | 50.9 | 61.1 | 71.6 | 52.9 | 28.6 |  |  |

*p-value for analysis from analysis of variance (ANOVA), ** p-value from chi-square

**Appendix Table 3. Rotated factor loading on health beliefs about smoking among those with a lifetime history of smoking**

| Health Beliefs | Factor 1 | Factor 2 | Factor 3 | Factor 4 |
| --- | --- | --- | --- | --- |
| Q1. You enjoy smoking too much to give it up | 0.8272 | 0.3732 | 0.0329 | -0.0992 |
| Q2. Smoking clams you down when you are stress or upset | 0.7147 | 0.1954 | -0.2850 | 0.0338 |
| Q3. Smoking helps you concentrate better | 0.8800 | 0.2286 | -0.0772 | 0.0209 |
| Q4. Smoking is an important part of your life | 0.8397 | 0.3035 | 0.0936 | 0.1151 |
| Q5. Smoking makes it easier for you to socialize | 0.6936 | 0.3889 | 0.2582 | -0.0478 |
| Q6. The medical evidence that smoking is harmful is exaggerated | 0.1086 | 0.5316 | -0.0715 | 0.2723 |
| Q7. Smoking is no more risky than lots of other things that people do | 0.2910 | 0.7707 | -0.0569 | 0.1164 |
| Q8. You have to die of something, so why not enjoy yourself and smoke | 0.5460 | 0.6617 | -0.0037 | -0.0986 |
| Q9. I think I must have the sort of good genes that means I can smoke without getting any harm | 0.5396 | 0.6664 | 0.1512 | -0.0943 |

**Appendix Table 4 Distribution of respondents to each of the functional beliefs and risk minimizing beliefs of smoking**

| Health Beliefs | Agreement score | | | | |
| --- | --- | --- | --- | --- | --- |
|  | 1 | 2 | 3 | 4 | 5 |
| Q1. You enjoy smoking too much to give it up: (n, row %) | 57 (30.5) | 48 (25.7) | 59 (31.6) | 13 (6.9) | 10 (5.3) |
| Q2. Smoking clams you down when you are stress or upset :(n, row %) | 36 (19.2) | 32 (17.1) | 56 (30.0) | 44 (23.5) | 19 (10.2) |
| Q3. Smoking helps you concentrate better: (n, row %) | 61 (32.6) | 36 (18.7) | 65 (34.8) | 16 (8.6) | 10 (5.3) |
| Q4. Smoking is an important part of your life: (n, row %) | 76 (40.6) | 46 (24.6) | 47 (25.1) | 8 (4.3) | 10 (5.3) |
| Q5. Smoking makes it easier for you to socialize: (n, row %) | 91 (48.7) | 43 (23.0) | 38 (20.3) | 8 (4.3) | 7 (3.7) |
| Q6. The medical evidence that smoking is harmful is exaggerated: (n, row %) | 32 (17.1) | 37 (19.8) | 36 (19.3) | 36 (19.3) | 46 (24.6) |
| Q7. Smoking is no more risky than lots of other things that people do: (n, row %) | 52 (27.8) | 46 (24.6) | 52 (27.8) | 19 (10.2) | 18 (9.6) |
| Q8. You have to die of something, so why not enjoy yourself and smoke: (n, row %) | 58 (31.0) | 48 (25.7) | 53 (28.3) | 15 (8.0) | 13 (7.0) |
| Q9. I think I must have the sort of good genes that means I can smoke without getting any  harm: (n, row %) | 70 (37.4) | 45 (24.1) | 54 (28.9) | 7 (3.7) | 11 (5.9) |

A score of five indicated that the participant totally agreed with the statement, a score of four indicated that the participant somewhat agreed with the statement, a score of three reflected that the participant was unsure about the statement, a score of two and a score of one indicated that the participant somewhat disagreed and totally disagreed with the statement

**Appendix Table 5. Rotated factor loading on health beliefs about alcohol among those with a lifetime history of alcohol drinking**

| Health Beliefs | Factor 1 | Factor 2 | Factor 3 | Factor 4 |
| --- | --- | --- | --- | --- |
| Q1. You enjoy drinking too much to give it up | 0.6017 | 0.5269 | 0.2458 | 0.0400 |
| Q2. Drinking clams you down when you are stress or upset | 0.4487 | 0.6586 | 0.1952 | -0.0118 |
| Q3. Drinking helps you concentrate better | 0.7478 | 0.3025 | 0.2209 | -0.0086 |
| Q4. Drinking is an important part of your life | 0.7560 | 0.3092 | 0.1409 | -0.0151 |
| Q5. Drinking makes it easier for you to socialize | 0.4014 | 0.6221 | 0.1853 | -0.0392 |
| Q6. The medical evidence that drinking is harmful is exaggerated | 0.1819 | 0.1846 | 0.4248 | -0.0843 |
| Q7. Drinking is no more risky than lots of other things that people do | 0.2953 | 0.2869 | 0.5219 | -0.0379 |
| Q8. You have to die of something, so why not enjoy yourself and drink | 0.4328 | 0.4947 | 0.4026 | 0.0916 |
| Q9. I think I must have the sort of good genes that means I can drink without getting any harm | 0.5012 | 0.4092 | 0.4413 | 0.0996 |

**Appendix Table 6 Distribution of respondents to each of the functional beliefs and risk minimizing beliefs of alcohol drinking**

| Health Beliefs | Agreement score | | | | |
| --- | --- | --- | --- | --- | --- |
|  | 1 | 2 | 3 | 4 | 5 |
| Q1. You enjoy drinking too much to give it up; (n, row %) | 770 (49.3) | 389 (24.9) | 276 (17.7) | 84 (5.4) | 4 (2.7) |
| Q2. Drinking clams you down when you are stress or upset; (n, row %) | 606 (38.8) | 293 (18.8) | 359 (23.0) | 235 (15.0) | 69 (4.4) |
| Q3. Drinking helps you concentrate better; (n, row %) | 948 (60.7) | 376 (24.1) | 179 (11.5) | 32 (2.0) | 27 (1.7) |
| Q4. Drinking is an important part of your life; (n, row %) | 1,019 (65.2) | 344 (22.0) | 153 (9.8) | 20 (1.3) | 26 (1.7) |
| Q5. Drinking makes it easier for you to socialize; (n, row %) | 600 (38.4) | 281 (18.0) | 376 (24.1) | 251 (16.1) | 54 (3.5) |
| Q6. The medical evidence that drinking is harmful is exaggerated; (n, row %) | 518 (33.2) | 295 (18.9) | 296 (19.0) | 247 (15.8) | 206 (13.2) |
| Q7. Drinking is no more risky than lots of other things that people do; (n, row %) | 597 (38.2) | 430 (27.5) | 331 (21.2) | 151 (9.7) | 53 (3.4) |
| Q8. You have to die of something, so why not enjoy yourself and drink; (n, row %) | 734 (47.0) | 377 (24.1) | 278 (17.8) | 105 (6.7) | 68 (4.4) |
| Q9. I think I must have the sort of good genes that means I can drink without getting any harm; (n, row %) | 761 (48.7) | 376 (24.1) | 332 (21.2) | 56 (3.6) | 37 (2.4) |

A score of five indicated that the participant totally agreed with the statement, a score of four indicated that the participant somewhat agreed with the statement, a score of three reflected that the participant was unsure about the statement, a score of two and a score of one indicated that the participant somewhat disagreed and totally disagreed with the statement
