# Supplementary figures and images for: Functional beliefs and risk minimizing beliefs among Thai healthcare workers in Maharaj Nakorn Chiang Mai hospital: its association with intention to quit tobacco and alcohol
Source: Subst Abuse Treat Prev Policy. 2017 Jul 12;12:34. doi: 10.1186/s13011-017-0118-1 (PMC5508689; doi:10.1186/s13011-017-0118-1)

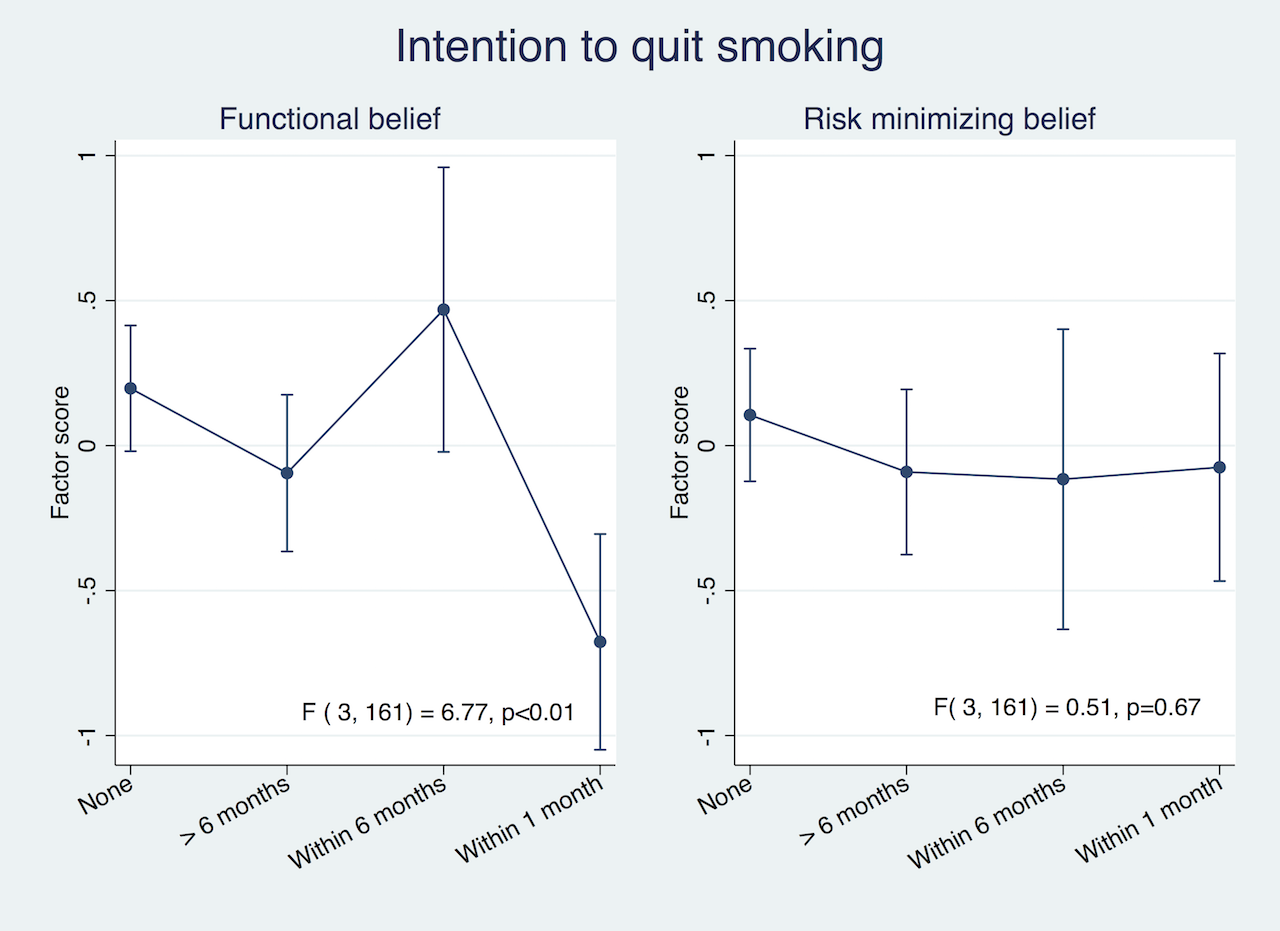

Supplement: Supplementary file 2 — Sensitivity Analysis of Health Beliefs and Intention to Quit Smoking (excluding recent quitters). Results are adjusted for age and sex. Higher factor score indicate higher level/agreement of belief. Vertical lines represents 95% confidence intervals. P-values obtained from values of the F statistic and the corresponding degrees of freedom. (TIFF 4656 kb) [file 13011_2017_118_MOESM2_ESM.tiff]

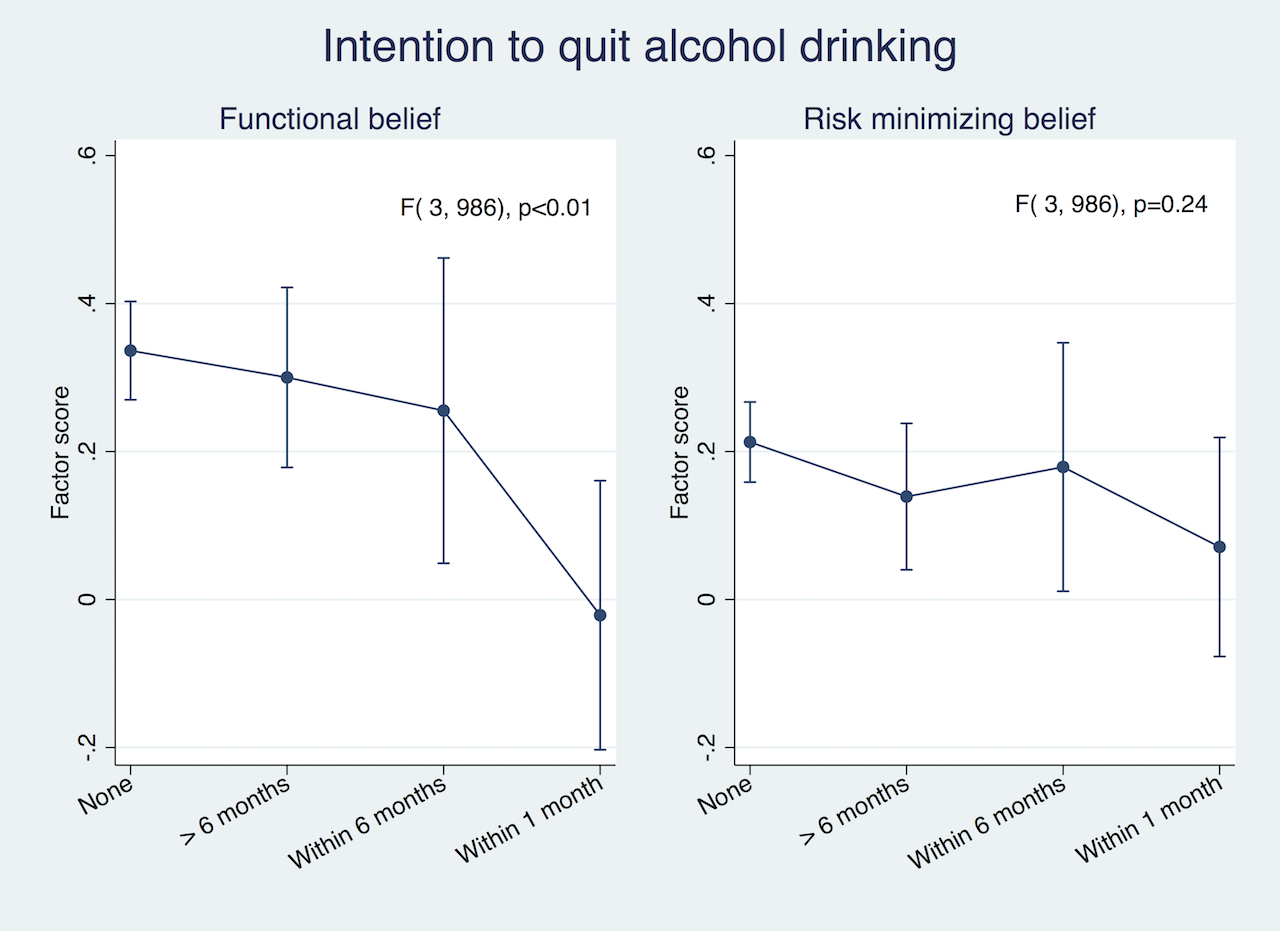

Supplement: Supplementary file 3 — Sensitivity Analysis of Health Beliefs and Intention to Quit Alcohol Drinking (excluding recent quitters). Results are adjusted for age and sex. Higher factor score indicate higher level/agreement of belief. Vertical lines represents 95% confidence intervals. P-values obtained from values of the F statistic and the corresponding degrees of freedom. (TIFF 4656 kb) [file 13011_2017_118_MOESM3_ESM.tiff]
